# Supplementary material for: EventLFM: event camera integrated Fourier light field microscopy for ultrafast 3D imaging
Source: Light Sci Appl. 2024 Jun 26;13:144. doi: 10.1038/s41377-024-01502-5 (PMC11199625; doi:10.1038/s41377-024-01502-5)
Supplement: Supplementary file 1 — Supplementary Information [file 41377_2024_1502_MOESM1_ESM.docx]

**Supplementary Information for EventLFM: Event Camera integrated Fourier Light Field Microscopy for Ultrafast 3D imaging**

Ruipeng Guo^1^, Qianwan Yang^1^, Andrew S. Chang^2^, Guorong Hu^1^, Joseph Greene^1^, Christopher V. Gabel^2,3^, Sixian You^4^, and Lei Tian^1,3,5,*^

^1^Department of Electrical and Computer Engineering, Boston University, Boston, MA 02215, USA.

^2^Department of Physiology and Biophysics, Boston University, Boston, MA 02215, USA.

^3^Neurophotonics Center, Boston University, Boston, MA 02215, USA.

^4^Research Laboratory of Electronics (RLE) in the Department of Electrical Science and Engineering, Massachusetts Institute of Technology, Cambridge, MA 02139, USA.

^5^Department of Biomedical Engineering, Boston University, Boston, MA 02215, USA.

^*^Correspondence: leitian@bu.edu, Tel.: 1-617-353-1334

Authors’ email:

Ruipeng Guo: [rguo@bu.edu](mailto:rguo@bu.edu)

Qianwan Yang: [yaw@bu.edu](mailto:yaw@bu.edu)

Andrew S. Chang: [aschang@bu.edu](mailto:aschang@bu.edu)

Guorong Hu: [grhu@bu.edu](mailto:grhu@bu.edu)

Joseph Greene: [joeg18@bu.edu](mailto:joeg18@bu.edu)

Christopher V. Gabel: [cvgabel@bu.edu](mailto:cvgabel@bu.edu)

Sixian You: [sixian@mit.edu](mailto:sixian@mit.edu)

Lei Tian: leitian@bu.edu

1. **Setup and system characterization**

The EventLFM system is a novel integration of a traditional Fourier light field microscope (LFM) and an event camera, as shown in Fig. S1. Our system consists of three primary optical paths: the illumination path, the Fourier LFM imaging path and the reference path.

**Illumination path**: A blue LED (SOLIS-470C, Thorlabs) is used as the excitation source for imaging GFP fluorescence. To maximize light efficiency, two condensers (CL1 and CL2) are used to collect and collimate the highly divergent LED source. Next, two field lenses (L1 and L2) are used to focus the beam onto the back pupil plane of the objective lens (Plan Apo, 20×, Nikon) and pass through a filter set to ensure uniform illumination within the target volume.

**DMD setup**: A DMD (DLI4130 0.7 XGA VIS High-Speed Kit, Digital Light Innovations) is set up in illumination path to manipulate the distribution of excitation in FOV. Beam is illuminated onto the face of DMD with a degree of 24^o^ through a TIR prism. Then the DMD face is imaged in the FOV with demagnification of ~40 by a 4F system and the objective. By designing the pattern on DMD, we can generate structured illumination as needed.

**Fourier LFM Detection Path**: Fluorescence emission from the sample is collected by the objective lens (Plan Apo, 20×, 0.75 NA, Nikon). A beamsplitter (BS028, Thorlabs) is placed after the filter set to split the fluorescence signals into the Fourier LFM and reference detection paths. For the Fourier LFM path, a tube lens (TL, *f* = 200 mm, ITL200, Thorlabs) projects the signal onto the focal plane of a Fourier lens (FL, AC508-080-A, Thorlabs), which performs a Fourier transform on the intermediate image. A microlens array (MLA, S600-f28, RPC photonics) is placed at the back focal plane of the FL to uniformly sample the angular information and generate a 5×5 array of elemental images. To efficiently utilize the sensor size, a 4f system is implemented after the MLA.

**Reference Path**: In parallel, the reference path employs a lens (LA1417-A, *f* = 150 mm, Thorlabs) and an sCMOS camera (CS2100M-USB, Thorlabs) to form a conventional widefield fluorescence microscope.

Figure S1: **Sketch of the EventLFM setup.**

**Integration of Event Camera**: Within the 4f system of the Fourier LFM path, an additional beamsplitter (BS013, Thorlabs) is introduced to distribute the light field onto both an event camera (EVK4, Prophesee) and an sCMOS camera (CS2100M-USB, Thorlabs). The 4f systems before the event camera and sCMOS camera share the same parameters. This configuration allows for simultaneous recording of dynamic fluorescence signals at identical magnifications, facilitating a direct and unbiased comparison between the EventLFM system and the traditional Fourier LFM.

We systematically assessed the performance characteristics of the traditional Fourier LFM system equipped with an sCMOS camera.

**Field of View (FOV) Calibration**: to determine the system’s FOV, we employed a calibration strategy using a single reference bead. The bead was translated across the imaging plane using a motorized stage. By examining the maximum range of displacements without exceeding the boundaries of each elemental images, we quantified the FOV to be approximately 130 μm, as illustrated in Fig. S2a.

**Depth of Field (DOF) Analysis**: Theoretically, our system’s DOF is approximately 300 μm, based on the shift limit of images originating from the outermost microlenses. However, we observed severe aberrations when the bead is moved beyond the central 200 μm depth range. Fig. S2b shows the images of a single bead at z-positions of -100 μm, 0 μm and 100 μm. Point spread function (PSF) aberrations are markedly evident at z-positions of ±100 μm from the focal plane. Consequently, we constrained the system’s operational DOF to 200 μm in this study.

Figure S2: **System characterization**. **a** Two frames captured from a single bead by the traditional Fourier LFM, showcasing the PSFs at leftmost and rightmost boundary of the FOV. **b** Three frames captured at three *z* positions, including -100 μm, 0 μm and 100 μm, using the same Fourier LFM system. Notable aberrations become evident at the -100 μm and 100 μm axial positions as illustrated in the zoom-in regions. **c** Depth color-coded map of a reconstructed frame from the EventLFM system and Fourier LFM system. **d, e** the intensity profiles along the x and z axis for the bead indicated by the arrow in **c**. **f** 3D MTF of the Fourier LFM system.

**Resolution Evaluation**: To evaluate the system’s resolution, we utilized a phantom consisting of 2 μm fluorescent beads. We employed the refocusing algorithm to achieve 3D reconstruction, resulting in a color-coded depth map of a reconstructed frame, as shown in Fig. S2c. Lateral and axial resolution metrics were extracted from the full-width at half-maximum (FWHM) measurements taken along profiles across a selected bead, as displayed in Fig. S2d for the *x* axis and Fig. S2e for the *z* axis. These measurements yielded a FWHM of 3.9 μm in the x dimension and 21.0 μm in the z dimension, findings that are in concordance with the 3D Modulation Transfer Function (MTF) shown in Fig.S2f.

1. **Detailed computational pipeline for EventLFM reconstruction**

We provide a detailed reconstruction pipeline for EventLFM in Fig. S3**.** Initially, the raw space-time event stream is processed via a time surface algorithm, effectively converting the dynamic event stream into frames. Following this conversion, a median filter is applied to the time surface frame, aiming to remove the discretized noise. Subsequently, the denoised frame is segmented into 5 x 5 views, with each view representing the viewpoint of an individual microlens. These views are shifted at subpixel resolution and cumulated in a series of refocusing images, each corresponding to a different focal depth. These refocusing images are then sequentially arranged into a volume with a 2 µm depth interval. The culmination of this process involves a thresholding technique, strategically applied to the refocusing volume to eliminate artifacts, thereby ensuring a high-quality reconstruction. Finally, the refocusing volume is then filtered by a threshold to mitigate the refocusing artifacts.

Figure S3: **Detailed computational pipeline**. **a** Space-time event spike stream captured by the event camera, where each event is characterized by its polarity, timestamp, and spatial coordinates. **b** The raw event stream is transformed into a single frame via the time surface algorithm with an integration time of 1 ms. **c** The time surface frame is denoised via a medium filter to enhance the SNR. **d** The denoise frame is cropped into 5x5 views, which are then realigned and cumulatively added (indicated by the orange arrow) to form the refocusing planes at different depths. **e** The refocusing volume is a sequence of 100 refocused planes with a 2 µm depth interval. **f** The refocusing volume is filtered via a threshold to reduce the artifacts.

1. **Time-surface algorithm**

The notion of a “time surface” is a concept used in the domain of event cameras, serving to enrich the representations for recognition tasks [1, 2]. In this context, an algorithm is employed to harness an exponential time-decay function, thus generating a time surface that encapsulates both the spatial and temporal correlations existing among adjacent pixels. Subsequently, the pixel value comprising this surface, signifying the signal intensity, are assigned values ranging from 0 to 255, as shown in Fig. S4a. When a pixel is selected as the most recent event, all other pixels within the temporal stream undergo value assignment contingent upon their respective time-stamps and the decay curve, with earlier events being ascribed smaller values. This approach offers a spatiotemporal context for each specific event, thereby effectively minimizing undesirable motion blurs.

Figure S4: **Time-surface algorithm**. **a** Exponential decay curve used in time-surface algorithm. **b** Visual comparison between directly-accumulated frames and time-surface frames. Top row: 1 ms accumulation time. Bottom row: 5 ms accumulation time. **c** The color-coded depth maps of reconstructed frame generated through the directly-accumulated frames with accumulation times ranging from 1 ms to 10 ms. **d** Color-coded depth maps of reconstructed frames using time-surface frames with accumulation times ranging from 1 ms to 10 ms.

Besides the time-surface algorithm, an alternative method of translating events stream into conventional image frames is the direct accumulation of all events within a defined temporal window into a single frame. A visual comparison of outcomes resulting from the time-surface algorithm and the direct event accumulation approach for a fast-moving object is provided in Fig. S4b. When an accumulation time of 1 ms is employed, both methods yield comparable results. However, when a longer accumulation time is employed, the time-surface method yields frames where motion trails appear less pronounced. Similarly, for dynamic signals with varying velocities processed using specific accumulation times, the time-surface method excels at suppressing motion-induced blurs in the resulting frames. We further perform light field reconstructions utilizing data from both directly accumulated frames and time-surface frames, the resulting volumes represented in color-coded depth maps are shown in Fig. S4c-d. For this experiment, the speed of the object is set at 2.5 mm s^-1^. When employing a lower accumulation time, both methods yield similar results. However, with an increase in the accumulation time, the efficacy of the time-surface algorithm in mitigating motion blur artifacts (Fig. S4c) becomes apparent as compared to the direct-accumulation method (Fig. S4d). This demonstrates the superior capability of the time-surface algorithm for processing event streams from dynamic objects.

1. **Additional details of denoising process and noise analysis**

**Details of the denoising process for biological samples**:

The denoising efficiency of our reconstruction algorithm hinges on two critical operations: the median filter and the summing operation within the refocusing algorithm. Fig. S5 illustrates the impact of these operations on noise reduction during the imaging of neuron-labeled brain slices.

Figure S5: **Detailed denoising process for brain slice**. The first and second rows compare the refocusing results without and with a medium filter. The medium filter effectively removes the discretized noise from the highly sensitive event sensor. Further enhancement of SNR is achieved through the shift-and-sum refocusing algorithm. The refocusing results from EventLFM are validated with traditional Fourier LFM. The white arrows indicate how different neurons are focused on different depths. The comparison with Fourier LFM shows that the EventLFM can accurately reconstruct depth information and suppress the background noise without sacrificing spatial resolution.

Initially, the raw data's time surface is heavily overwhelmed by the sensor noise (shown in the first row of Fig. S5), applying a median filter effectively removes a substantial amount of the noise while preserving the neuronal structures. This efficiency stems from the inherent characteristics of event cameras, which, unlike traditional sensors that capture images at predetermined intervals, asynchronously detect illumination changes at the pixel level. This process naturally leads to a more ‘discretized’ form of noise, which can be mitigated via medium filtering. Subsequent enhancement of the signal-to-noise ratio (SNR), by approximately 5X, is facilitated by the summing of 25 aligned cropped views within the refocusing algorithm. This enhancement stems from the fact that noise across different views is *uncorrelated*, which permits the summation process to effectively reduce noise while amplifying the signal of in-focus neuronal structures.

Validation of the reconstructed images is achieved through comparison with a conventional Fourier LFM system. The alignment of white arrows in both Fourier LFM and EventLFM, highlighting neurons at various refocusing depths, demonstrates the ability of the reconstruction pipeline to accurately recover neuronal depth information. Furthermore, the event camera's inherent sensitivity to brightness variations provides an intrinsic advantage in suppressing out-of-focus blur and background noise. Consequently, this leads to a cleaner background and higher spatial resolution compared to conventional Fourier LFM.

**Noise analysis for EventLFM with different sensitivity**:

We follow a similar procedure in [4] to characterize the noise characteristics in EventLFM. First, electronic noise is tested when the EventLFM is in a dark environment. Initially, we record the event stream with three different sensitivity thresholds. Then we accumulate all the noise events together and calculate the number of events per pixel and per second as shown in Fig. S6a. Second, we quantify photon noise by measuring the random noise with a constant illumination onto the event sensor. To quantify the photon noise, a square light source, with an intensity approximating neuronal fluorescence signal, is employed as a constant illuminator. We accumulate the photon noise events within the illuminated square and calculate the number of events per pixel per second as shown in Fig. S6b. Photon noise dominates over electronic noise at high sensitivity setting, which is the sensitivity setting we used in this study. The noise is mainly isolated pixels randomly distributed across the FOV. We can use a medium filter to mitigate them. In addition, the refocusing algorithm is beneficial in noise suppression.

We evaluate the noise suppression effect of our algorithm by processing the recorded stream from a blinking square uniform source. We calculate the mean as the signal and the standard deviation (std) as the noise, and signal-noise-ratio (SNR) is defined as the ratio between the mean and the std. Fig. S6c illustrates the SNR of the raw images, the median-filtered images, and refocused images across three sensitivity settings, corroborating the enhancement of SNR through both the median filter and the refocusing algorithm.

Figure S6: **Noise analysis for EventLFM**. **a** Events induced by electronic noise are obtained in complete darkness. **b** Events induced by photon noise are recorded under constant illumination. **c** SNR comparison across raw image, processed image with medium filter, and refocused image with refocusing algorithm at three different sensitivity settings. Medium filter and refocusing algorithms both improve the SNR.

**Selection of event camera setting:**

The response threshold is a balance between sensitivity and background noise, as shown in Fig. S6. Reducing the threshold improves the pixel sensitivity, and increases pixel response speed, while also inducing more background noise. The selection of an appropriate response depends on the application's demands, with applications requiring a faster response time and detection of subtler brightness changes necessitating a lower threshold. We set it to ensure sufficient sensitivity at practical fluorescence imaging conditions in this work. The integration time directly determines imaging speed. The shorter the integration time is set, the faster the system could achieve. On the other hand, shorter integration time means lower signal. So, we set the response threshold lower to improve the pixel sensitivity and increase pixel response speed, which also induces more background noise and result in low SNR. This highlights the unique challenge in this work that achieves kHz 3D imaging under low SNR situation. The main criterion is to ensure high quality imaging under low SNR.

1. **Comparison between EventFLM and traditional Fourier LFM for imaging fast-moving objects**

Figure S7: **Comparison between EventLFM and Traditional Fourier LFM for imaging fast moving objects. a** Color-coded depth map of reconstructed frames from the same object moving at varied speeds, captured by the traditional Fourier LFM equipped with an sCMOS camera. **b** Color-coded depth map of the reconstructed frame from the same object moving at 2.5 mm s^-1^, acquired with EventLFM.

To elucidate on the superior capability of EventLFM in capturing fast-moving objects, we conduct the following controlled experiments to benchmark the EventLFM’s result with the traditional Fourier LFM. First, to demonstrate the performance of the traditional Fourier LFM for imaging moving object, we conduct a series of tests using 3D phantoms moving at various speeds. Initially, the object was imaged while at rest, and reconstruction was performed by employing the standard light field refocusing algorithm, serving as a benchmark. Subsequently, the object’s speed was set at 0.2 mm s^-1^, and a sequence of frames was captured for subsequent reconstruction. Following this, the speed was adjusted to 2.5 mm s^-1^, and the same region of the sample was imaged. Upon completion of the reconstruction process, frames containing identical region of interest were selected to generate the color-coded depth map, as depicted in Fig. S7a. The MIPs obtained when the object was stationary and at a speed of 0.2 mm s^-1^ displayed close agreement, validating the consistency of the imaging process. However, when the object’s speed increased to 2.5 mm s^-1^, the reconstructed results exhibited considerable motion blur artifacts. The artifacts can be attributed to the constrained frame rate (30 fps) of the sCMOS camera employed. To demonstrate the unique capability of EventLFM for imaging fast-moving object. simultaneous imaging of the object moving at 2.5 mm s^-1^ was conducted with an event camera. Subsequently, reconstruction was performed by employing the same refocusing algorithm, as depicted in Fig. S7b. Remarkably, the reconstruction closely matched the results obtained with the sCMOS camera under slow-moving conditions.

1. **Imaging of moving object in z direction**

In addition to evaluating the performance of EventLFM for objects moving along the y-axis, we extend our assessment to include objects moving along the *z* axis. Fig. S8 illustrates the relationship between the accumulation time and the quality of the reconstructed images. An increase in accumulation time results in a decrease in resolution, with the emergence of a tail artifact attributable to the object's high velocity. For this experiment, we utilize a phantom moving along the *z* axis at a controlled speed of 0.9 mm s^-1^, a rate dictated by the working distance limitations of our setup. We process the stream with varying accumulation times, ranging from 5 ms to 40 ms. Fig. S8 presents the MIPs of the reconstructed images in both the *x-y* and *y-z* planes. Consistent with observations from the phantom moving in the *y* direction, we note that the apparent size of the reconstructed beads expanded with increasing accumulation time, and a pronounced tail appears at higher accumulation time.

Figure S8: **Reconstructed results of phantom moving in *z* direction.** With the same recorded stream, we use two different accumulation time: 1 ms and 5ms and calculate the MIPs in both x-y plane and y-z plane.

1. **Comparison between EventFLM and traditional Fourier LFM for imaging neuron labeled *C. elegans***

In order to assess the applicability of our EventLFM system for biological specimens, we conducted imaging of GFP-labeled neurons within multiple freely moving *C. elegans*. These *C. elegans* were positioned on a gel substrate and subsequently exposed to a droplet of Basel solution. Within the *C. elegans* body, four brightly labeled neurons are present, with two located in the tail region and two in the mid-body section. When observed through a fluorescence microscope, these neurons are readily visible. However, by increasing the accumulation time of the event camera, we were able to discern the vague outline of the *C. elegans* body, as depicted in Fig. S9a. Dashed lines approximately delineate the positions of the *C. elegans* within the FOV. Signals from the *C. elegans* were simultaneously captured using both LFM systems using the event camera and an sCMOS camera, resulting in reconstructions presented in Fig. S9b. Since the event camera only detects moving or blinking signals. Consequently, the two neurons at the bottom, which remained relatively stationary during recording, are largely absent from the reconstruction obtained from the event camera. For a more detailed examination, we conducted a zoomed-in analysis and traced the trajectories of the four neurons within the central area, as illustrated in Fig. S9c. This analysis distinctly reveals the movement of the neurons along their respective trajectories within the 3D space.

Figure S9: **Additional imaging results of freely-moving *C. elegans*.** **a** An example of a *C. elegans* specimen used in our experiment captured by an event camera. The dashed line delineates the contour of *C. elegans* bodies. **b** Comparison of color-coded depth maps from the reconstructed volumes using EventLFM and the traditional Fourier LFM. **c** 3D zoom-in reconstructions and the tracked trajectories of neurons from EventLFM.

1. **More results from blinking neurons in mouse brain slice**

To demonstrate EventLFM’s potential for neural imaging, we image a 75 µm thick section of GFP-labeled mouse brain tissue. The sample is illuminated using a pulsed LED source, designed to simulate neuronal activities within scattering biological tissues. The illumination pulse sequence is set with a 1 ms pulse width and intervals varying from 2 ms to 50 ms. To validate the spatial reconstruction accuracy of EventLFM, we capture the fluorescence signals with traditional Fourier LFM and conventional fluorescent microscopy under constant illumination. Fig. S10a shows MIPs from a single reconstructed frame of each method. By visual inspection, the reconstruction from EventLFM is consistent with Fourier LFM, effectively capturing all neurons within the FOV and the intensity variations among them. However, a notable difference arises in the signal-to-background ratio (SBR). Fourier LFM suffers from a low SBR due to tissue scattering, which results in neuronal signals being buried in strong background fluorescence. In contrast, EventLFM demonstrates a significantly improved SBR, yielding a reconstruction with markedly improved image contrast and suppressed background fluorescence. This improvement is attributed to the event-based measurement mechanism intrinsic to EventLFM, wherein a readout is generated only when intensity changes exceed a certain threshold. Consequently, temporally slowly varying background fluorescence signals, which do not often meet this criterion, are either removed or substantially reduced in the raw data. Additionally, to underline EventLFM’s capability of precisely recording fast neuronal spikes, temporal traces from three distinct neurons are extracted, as shown in Fig. S10b. These traces exhibit a strong correlation with the input illumination pulse sequence, thereby validating that EventLFM can accurately reconstruct neuronal blinking dynamics within scattering tissue.

Figure S10: **EventLFM imaging of blinking neurons in mouse brain tissue. a** MIPs of single-frame reconstructions, from EventLFM and Fourier LFM, capturing blinking neuronal signals within a 75 µm-thick mouse brain slice emulated with pulsed LED illumination. **b** Temporal traces from three distinct neurons, obtained by calculating the mean intensities in the dashed rectangles labeled 1, 2, and 3 in **a**. The LED pulses are set at 1 ms width, with the intervals varying randomly from 2 ms to 50 ms. The reconstructed traces agree with the LED pulse sequence, validating EventLFM’s ability to accurately capture blinking dynamics within scattering brain tissue.

1. **Additional details on imaging brain slice with target illumination**

**Hardware setup**:

A DMD (DLI4130 0.7 XGA VIS High-Speed Kit, Digital Light Innovations) is set up in illumination path to manipulate the distribution of excitation in the FOV. Beam is illuminated onto the surface of the DMD with a degree of 24^o^ through a TIR prism. The DMD surface is imaged onto the FOV with a demagnification of ~40 by a 4F system and the objective. By designing the pattern on the DMD, we can generate spatiotemporal illumination as needed.

**Pattern generation**:

First, we place a fluorescent plate on stage and use the DMD to display a designed pattern. The fluorescence excited by the DMD pattern is captured by the reference camera. Utilizing the *imtransform* function within MATLAB, a transformation matrix is extracted, establishing a correspondence between a designated DMD pattern and its associated image. Then a brain slice replaces the fluorescent plate in the FOV and the reference camera captures the neurons in the FOV under uniform illumination. By employing the transformation matrix, a corresponding DMD pattern is generated, specifically tailored to the neurons of interest within the brain slice. Individual patterns from the neurons are isolated into separate frames. These frames are then integrated into a sequence at varying intervals. Upon activation of the DMD, it projects this sequence of patterns, thereby achieving targeted illumination of the predetermined neurons.

**Additional 3D reconstruction results**:

We provide an additional comparison of the 3D information of the mouse brain tissue detailed in manuscript Section 3.3. Fig. S11 compares the axial profile along the dashed line among axial scanned widefield measurement with uniform and targeted illumination, and EventLFM with targeted illumination. The axial displacement of neurons from EventLFM imaging results are consistent with the widefield system, demonstrating EventLFM’s capability to capture 3D information from the brain slice tissue. Moreover, targeted illumination significantly suppresses background noise originating from out-of-focus regions or scattered signals from other neuronal structures, which substantially improves both the SNR and SBR for neuronal activity detection.

******

Figure S11: Comparison of the XY MIP and z profile traced along the dashed red and yellow lines across the axial scanned widefield measurement with uniform illumination and targeted illumination, and EventLFM with targeted illumination.

1. **Convolutional Neural Network for EventLFM reconstruction.**

To further demonstrate the potential imaging capabilities of EventLFM, we have integrated a deep learning framework tailored for 3D reconstruction. Preliminary results with fast-moving and dynamic-blinking phantoms embedded with fluorescent particles demonstrate that EventLFM augmented by deep learning facilitates significant improvements in 3D resolution and overall reconstruction quality.

**Network structure**:

Our EventLFM-Net draws inspiration from the CM^2^Net, which is tailored for high-resolution volumetric reconstruction in imaging systems with multi-view geometry [3]. The network contains two main modules: the view synthesis module and the LFR enhancement module (shown in Fig. S12). The view synthesis module extracts the subpixel parallax information directly from the raw view stack, while the LFR enhancement module enhances upon the initial refocusing volume. Collectively, the network effectively suppresses the reconstruction artifacts from the ‘shift-and-add’ algorithm and enhances the 3D resolution.

Figure S12: **EventLFM-Net structure**. The input of EventLFM-Net consists of a cropped 5 x 5 view stack alongside the refocusing volume. The view stack is sent to a view synthesis module to integrate the information from multiple lenses. Concurrently, the refocusing volume is sent to an enhancement module to suppress the artifact and improve the 3D resolution from the refocusing volume. The two modules together yield a reconstructed volume with improved uniformity and resolution. The loss is performed by comparing the reconstructed volume with the widefield scanned ground truth.

Both modules have the same backbone structure, containing 16 Res-Blocks with additional skip connections to facilitate multi-scale feature fusion. The loss function is a sum of the Normalized Pearson Correlation Coefficient (NPCC) and Mean Absolute Error (MAE). This dual loss leverages NPCC to refine spatial alignment and MAE to minimize the intensity difference between the 3D reconstruction and ground truth, thus ensuring a comprehensive optimization of both spatial and intensity accuracy. The network is implemented with PyTorch and runs on an Nvidia GPU RTX 4090, with a batch size of 8. The entire training process takes 24 hours.

**Training data collection**: We utilize a phantom with a thickness of 200 μm to build the training dataset. First, we set the speed of the stage as 2.5 mm s^-1^ and recorded the stream with EventLFM when the sample is moving in y-direction. Then we move the sample back to the starting position and scan the sample in z direction step by step with the reference conventional wide-field microscopy (20X, 0.75 NA). The depth range is 210 μm with a scanning step size of 3 μm. The sample is moved to the next y position when the z-scanning is done. It takes about 4 hours to scan the whole area of the sample that is recorded by the event camera. We can crop the ground truth according to the refocusing results of the stream from the camera. In total, we collect a training dataset containing 4700 pairs of event stream and wide field ground truth.

**Blinking result on dynamic blinking object**:

The network trained on fast-moving objects is subsequently evaluated with a dynamic blinking object. The experiment setup is detailed in the manuscript Section 3.2. Fig. S13a presents the depth-color-coded RFV and CNN reconstruction of frame #1. Consistent with the results from fast-moving objects, the CNN reconstruction of the blinking object also outperforms RFV, showcasing enhanced resolution with improved image quality.

It is noteworthy that the event-based signal characteristics of blinking objects are distinct from those associated with fast-moving objects, as detailed in the manuscript. Despite these differences, by utilizing RFV as a preliminary estimation and conditioned on the network, our trained CNN can generalize across both moving and blinking phantoms. Additionally, Fig. S13b illustrates the temporal trace of three particles within the reconstruction, closely matching with the sequence of LED illumination pulses. This analysis confirmed that the network efficiently captures high-frequency blinking signals in a 3D context at enhanced resolutions, demonstrating its versatility and effectiveness in diverse dynamic imaging scenarios.

Figure S13: **Imaging results of a dynamic blinking object**. **a** Depth color-coded RFV and 3D CNN reconstruction of a frame marked as Frame #1. The CNN robustly generalizes to dynamic blinking objects, yielding improved image quality and enhanced resolution compared to RFV. **b** Temporal trace analysis for three particles, labeled 1, 2, and 3, within the CNN 3D reconstruction. The LED pulse widths are uniformly set at 1 ms with the inter-pulse intervals randomly varying between 2 ms and 50 ms.

1. **Analysis of the integration time choice in event data processing**

Event cameras differ from traditional cameras by operating *without* a fixed frame rate. Each pixel in an event camera functions asynchronously, with a timestamp resolution of 1 µs. The frame rate for EventLFM is determined *post hoc*, based on a “user-defined” integration time for accumulating events. This allows for *adjustable* frame rates during the post-processing of a recorded event stream. To assess the effect of the integration time, we conducted experiments using EventLFM system to capture an event stream from a moving disk with 75 µm in diameter. The event stream is then accumulated into event frames with varying integration times, as illustrated in Fig. 14b. We extracted a moving square ROI to compute the mean as the *signal* and the standard deviation as the *noise*. Subsequently, we analyze the relationship between the Signal-to-Noise Ratio (SNR) and integration time $\Delta t$, as depicted in Fig. S14c. The curve indicates a rapid decrease in SNR when the integration time is less than 1 ms, attributed to a decrease in the number of events as the integration time shortens. Intuitively, this is because the event detection is primarily limited by photon noise that follows the Poisson statistics (see Fig. S6). At a low integration time, the SNR decreases since fewer events are being accumulated. The curve shows that SNR approximately scales with $\sqrt{\Delta t}$. One should note that the event detection is based on the temporal change of log intensity exceeding a pre-defined threshold, which complicates the exact relationship between the raw photon count, SNR, and integration time.

Figure S14: **SNR vs Integration time**. **a** An example of a recorded event stream using EventLFM. **b** Events are accumulated into frames with varied integration time from 0.4 ms to 4 ms. **c** SNR vs integration time.

We further demonstrate the effect of integration to time EventLFM reconstruction results. As shown in the depth color-coded MIPs shown in Fig. S15, we process one recorded event stream from a moving object with varying integration times ranging from 0.2 ms to 8 ms to generate frames and reconstruct the object. When the integration time is below 0.4 ms, the fidelity of the reconstructed images is compromised due to an insufficient number of events being accumulated for accurate reconstruction. However, as the integration time increases, the images can be reconstructed with high fidelity, although artifacts such as tails may appear when the frame rate is not sufficient to capture the fast motion. Additionally, we captured an image using traditional FLFM when the object is stationary as a reference, which correlates well with the results from EventLFM. By adjusting the integration time in post-processing, the system can be tailored to suit various scenarios.

Figure S15: **Imaging results of a dynamic object with varied integration times**. The event stream is accumulated into frames with varied integration times ranging from 0.2 ms to 8 ms. As a reference, the same object is imaged with traditional FLFM when stationary.

1. **Comparison of event cameras and traditional high-speed cameras**

To showcase the advantages of event cameras over traditional high-speed cameras, we present a comparison in Table 1 with several commercially available high-speed cameras. The parameters listed for each camera reflect the maximum performance specifications from their datasheets. For event cameras, the maximum achievable frame rate is 10 kHz, constrained by a pixel latency of 100 µs. Both pixel latency and maximum event rate are sourced directly from the event camera datasheets. For traditional high-speed cameras, the data rate is calculated with the following equation:

$$Data rate=resoltuion\times frame rate\times bit depth$$

Firstly, traditional ultrafast cameras are significantly more expensive than event cameras, leading to higher overall costs for the imaging systems. Secondly, while traditional ultrafast cameras can deliver high data rates with high resolution and frame rates, they pose substantial challenges in data transmission and storage. The synchronous operation of CMOS or CCD sensors necessitates continuous frame-based storage, where just a single minute of recording can generate tens of gigabytes of data. This large data output requires external Solid State Drives (SSD) for onboard storage or advanced high-bandwidth transmission techniques to transfer data to host computers. These demands render ultrafast cameras less suitable for long-term monitoring of dynamic biological samples. Furthermore, given the sparsity of these biological samples, much of the continuously recorded data is redundant. For example, the ratio of pixels with effective information to the whole image in the brain slice shown in Fig. S11 is only ~12.5%. In contrast, event cameras operate asynchronously, detecting only changes within the scene. This significantly reduces data redundancy, thereby easing the burden on storage and transmission, allowing for capturing dynamic events over extended periods without interruption. Additionally, unlike fixed frame rate settings during recording for traditional cameras, event cameras capture an event stream and offer the flexibility to set any frame rate (below the limit set by the event camera’s latency), as discussed in Section 11.

**Table 1: Comparison of event camera and traditional ultrafast camera**


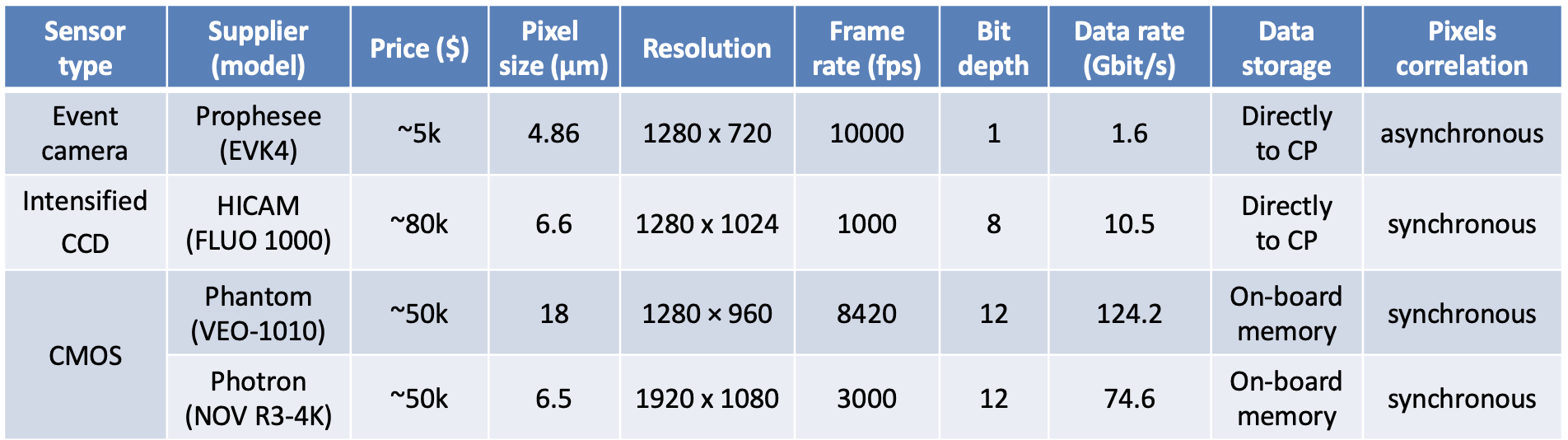


1. **Visualization 1 (Separate file)**

The video shows the motion of beads with different directions and speeds. The color represents the depth information. A transition in color corresponds to bead displacement in the z direction. The frame rate of the video is slowed down to 30 fps for visualization, though it was originally captured at a rate of 1000 fps.

**References**

1. Lagorce, X. et al. HOTS: A Hierarchy of Event-Based Time-Surfaces for Pattern Recognition. *IEEE Trans. Pattern Anal. Mach. Intell*. **39**, 1346–1359 (2017).
2. Sironi, A. et al. HATS: Histograms of averaged time surfaces for robust event-based object classification. *Proceedings of the IEEE conference on computer vision and pattern recognition* 1731–1740 (2018).
3. Xue, Y. et al. Deep-learning-augmented computational miniature mesoscope. *Optica* **9**, 1009 (2022).
4. Cabriel, C. et al. Event-based vision sensor for fast and dense single-molecule localization microscopy. *Nat. Photonics* **17**, 1105–1113 (2023).
